# The change of first metatarsal head articular surface position after Lapidus arthrodesis

**DOI:** 10.1186/s12891-018-2262-9

**Published:** 2018-09-27

**Authors:** Jan Klouda, Rastislav Hromádka, Simona Šoffová, Stanislav Popelka, Stanislav Popelka, Ivan Landor

**Affiliations:** 1 Department of Orthopaedics, Hospital Nemocnice České Budějovice, a.s., České Budějovice, Czech Republic; 2 Department of Orthopaedics, First Faculty of Medicine, Charles University and Motol University Hospital, Prague, Czech Republic; 3 Institute of Anatomy, First Faculty of Medicine, Charles University, Prague, Czech Republic

**Keywords:** Hallux valgus, Lapidus arthrodesis, Akin osteotomy, Tangential angle to the second axis, Proximal articular set angle, X-ray analysis

## Abstract

**Background:**

The Lapidus procedure has been used for hallux valgus deformity correction since 1931. In some cases, the arthrodesis results in an unfavourable lateral inclination of first metatarsal head articular surface. The objective of our study was to evaluate the change of orientation of this articular surface in relation to the second metatarsal axis by comparing pre- and postoperative radiographs. The secondary target was to evaluate possible benefits of combination of Lapidus and Akin procedures in the reduction of hallux valgus deformity.

**Methods:**

We evaluated 449 pre- and postoperative radiographs of 134 operations from 2010 to 2015. Routinely used angle measurements were performed on all X-rays. A sum of tangential angle to the second axis and distal articular set angle values was chosen as the best indicator for the deformity correction success.

**Results:**

The mean value of these angles total was 5.2° ±9.3° before and 14.2° ±7.8° after the operation. In the group of patients, where the additional Akin osteotomy was used, the mean value was 5.3° ±8.4° before and 6.9° ±10.2° after the surgery. The mean difference in values between the two groups (with and without Akin procedure) was 7.3° of extra correction in favour of the group with the Akin osteotomy.

**Conclusions:**

The mean worsening of the tangential angle after Lapidus operation was 6.1° ±6.9°, which counts for significant deterioration after a surgery. The Akin osteotomy was found to be a valuable addition to the Lapidus arthrodesis, which improves the position of articular surfaces in first metatarsophalangeal joint.

## Background

Hallux valgus deformity has been attracting attention of orthopaedic surgeons from all over the world over the last 150 years. An abundance of reconstruction techniques has been described in the history and forefoot reconstruction surgery remains in the limelight even in the modern era of orthopaedics.

One of techniques routinely used for operative treatment of moderate to severe hallux valgus deformity with first metatarsocuneiform (MTC) joint hypermobility is the surgical procedure originally described by Albrecht and popularized by Lapidus [1–3]. The procedure allows a very strong reduction of the deformity and a distinct change in position and rotation of the first metatarsal. Recent studies performed by Dayton et al. [4–7] and other authors [8–10] indicated that the rotational component of the deformity is key in its proper reduction.

The Lapidus procedure reduces first intermetatarsal angle (IMA) and changes the inclination and rotation of the first metatarsal head – that’s how it indirectly changes the position of the distal articular surface of the first metatarsal. The proper alignment of the surface is important for the correction of the deformity. The position of the surface can be measured on weight-bearing radiographs pre- and postoperatively [11, 12].

The objective of our study was to evaluate the change of orientation of the distal articular surface of first metatarsal in relation to the second metatarsal axis by comparing pre- and postoperative radiographs. The study evaluates influence of the derotation of the first metatarsal during the Lapidus procedure. The tangential angle to the second metatarsal axis (TASA) was used as a tool for description of the change of first metatarsal articular surface position.

A secondary goal of our study was to find out, if TASA measurement is a useful tool in the preoperative setting.

The third target of our investigation was to evaluate the possible benefits of combination of Lapidus and Akin procedures in the reduction of hallux valgus deformity.

## Methods

We retrospectively evaluated preoperative and postoperative radiographs of 134 feet (69 left, 65 right) of 110 patients (99 female, 11 male) who underwent the forefoot reconstruction surgery in Department of Orthopaedics, First Faculty of Medicine, Charles University in Prague at Motol University Hospital from 2010 to 2015. The mean age was 60.6 years (range 14 to 79). From the total of 110 patients, 19 patients (17%) had forefoot reconstruction surgery performed on both feet (not in one session). All patients were operated by 6 different surgeons.

The Lapidus procedure was performed from open medio-dorsal approach to the first MTC joint and the arthrodesis was fixed with 2–3 screws or 2 memory staples, in one case a plate was used for fixation during a revision surgery. The procedure was combined with other ones like Akin osteotomy or procedures on lesser toes. We used either open, miniinvasive or a combination of these approaches to the lesser toes and proximal phalanx of the hallux. The lateral release of first metatarsophalangeal (MTP) joint and medial eminence adjustment was done in all cases. The release was usually performed through longitudinal open approach in first web space or with McGlamry elevator through medial approach to the joint.

In every case, a preoperative weight-bearing X-ray (or X-rays) and a series of postoperative radiographs were obtained and separately evaluated by 2 investigators. The investigators measured totally 449 radiographs. An average set of radiographs for a case consisted of 3.3 X-ray pictures.

**Fig. 1:**
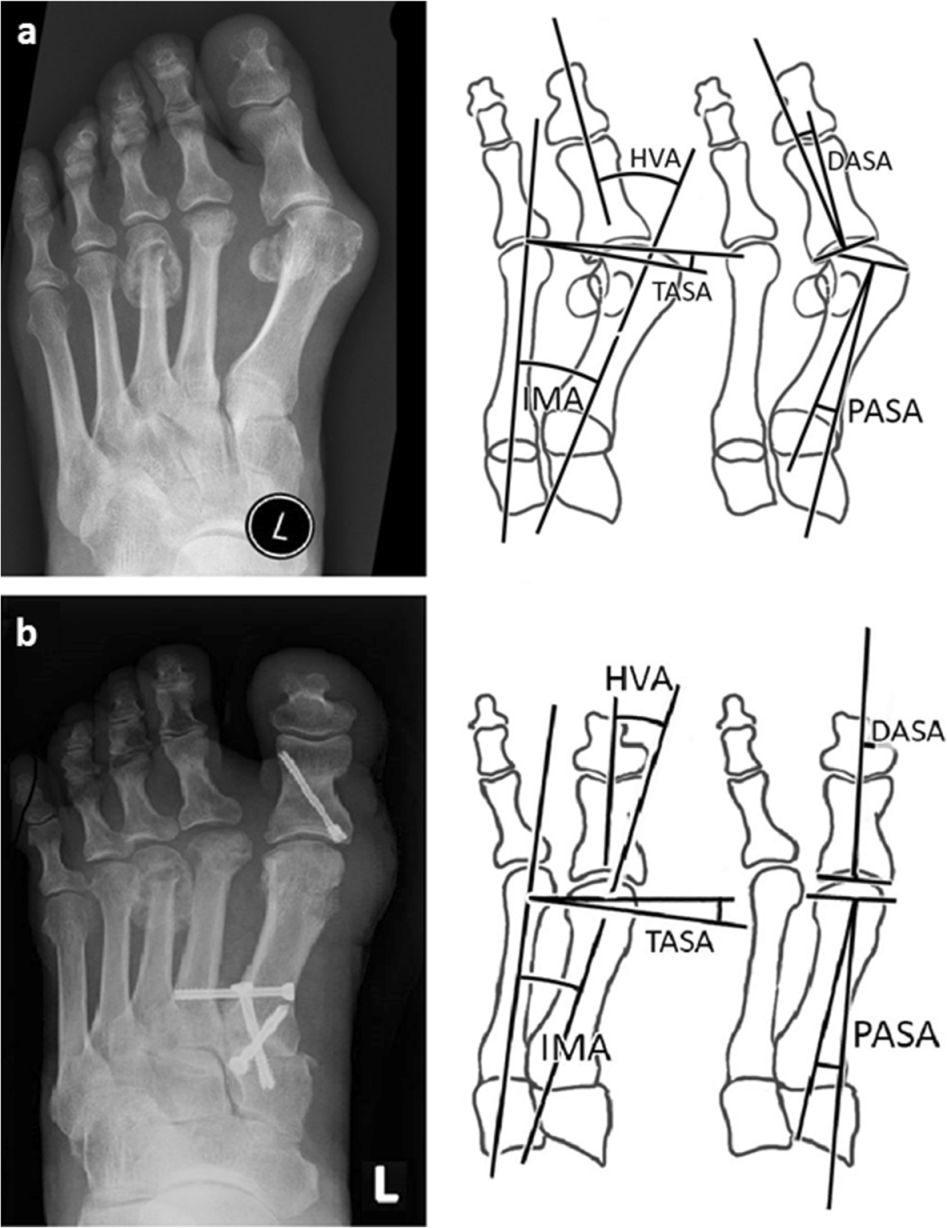
**a**, **b** Pre- and postoperative dorsoplantar radiographs of a patient who underwent Lapidus and Akin procedure. *Measurements of the HVA, TASA, DASA, PASA, and IMA. HVA – hallux valgus angle; TASA – tangential angle to the second axis; DASA – distal articular set angle; PASA – proximal articular set angle; IMA – intermetatarsal angle*

Two independent investigators assessed all X-rays using graphic analysis software (ImageJ). The first investigator (JK) was one of the surgeons, the other one (SS) was a clinical anatomist (Institute of Anatomy, First Faculty of Medicine, Charles University in Prague). They were blinded to each other’s measurements. On each picture, they had drawn five lines that were used for measurements (Fig. 1a, b). Firstly, the axis of the first metatarsal was defined as axis of the bone shaft. Similarly, the axes of the second metatarsal and proximal phalanx of the hallux were drawn. Fourth line went through medial and lateral margins of the articular surface of the first metatarsal head. Fifth line connected medial and lateral margins of proximal articular surface of the basal phalanx of the hallux.

Five angles were then measured on each radiograph: *hallux valgus angle* (HVA) – the angle between long axes of the first metatarsal and proximal phalanx, *intermetatarsal angle* (IMA) – the angle between long axes of the first and second metatarsal, *tangential angle to the second axis* (TASA) – the angle between the line connecting the medial and lateral margin of the articular surface of the first metatarsal head and a line drawn perpendicular to the long axis of the second metatarsal, *proximal articular set angle* (PASA) – the angle between long axis of the first metatarsal and a line drawn perpendicular to the articular surface of the first metatarsal head, *distal articular set angle* (DASA) – the angle between the long axis of proximal phalanx of the hallux and a line drawn perpendicular to its proximal articular surface (Fig. 1a, b).

After obtaining the data from selected patients´ radiographs, a statistical analysis was performed. Data of all angle measurements were compared to evaluate interobserver reliability and the method of measurement. The standard protocol with Cohen’s kappa coefficient for interrater reliability evaluation was used.

We evaluated the change of TASA, HVA, IMA and DASA caused by the surgery. The tangential angle to the second metatarsal axis (TASA) is one of angles, that can be assessed [13]. The angle is measured between the articular surface of the first metatarsal head and the long axis of the second metatarsal. It describes the inclination (even the rotation) of the articular surface in relation to the axis of the forefoot.

The TASA angle was chosen due to the type of surgery – Lapidus procedure does not include a distal metatarsal osteotomy, therefore a significant change of the first metatarsal articular surface position in relation to its axis (PASA) was not expected in the process.

The study was focused not only on differences between values of the TASA angle, but even on combination of angles. The group of patients was divided into two subgroups – one where only Lapidus procedure was performed and second group, where a complementary Akin operation was added. The DASA angle described the level of the closing wedge Akin osteotomy of the proximal phalanx. The sum of TASA and DASA values was chosen with intention to compare the subgroups to describe the alignment of first MTP joint after surgery.

## Results

On the preoperative and postoperative radiographs, the hallux valgus angle (HVA), intermetatarsal angle (IMA), tangential angle to second axis (TASA) and distal articular set angle (DASA) were measured by two independent observers. There were no statistically significant differences found between the two observers´ measurements (*p = 0.083, η*^*2*^ *= 0.028)*.

Evaluating our correction comparing preoperative to postoperative radiographs, these are the mean angle values for the whole group of 110 patients, not distinguishing the surgery type (both observers´ measurements together):

Before the surgery: The mean HVA was 45.6° ± 20.5 (range 25.0° to 77.0°), the mean IMA was 16.9° ± 3,8° (range 8.5° to 28.5°), the mean TASA was 3.8° ± 8.5° (range − 17.0° to 26.0°), mean PASA was 20.5° ± 7.5° (range 3.3° to 40.0°) and the mean DASA was 1.5° ± 4.0° (range − 9.0° to 20.0°).

After the surgery: The mean HVA was 24.6° ± 14.4° (range 2.1° to 36.0°), mean IMA was 9.8° ± 5.3° (range − 2.0° to 27.8°), the mean TASA was 9.9° ± 6.9° (range − 5.6° to 30.3°), mean PASA was 19.4° ± 6.9° (range 2.6° to 36.9°) and DASA 3.4° ± 5.2° (range − 13.5° to 15.0°). The summary of results is shown in Table 1.

**Table 1:** Angles of interest measured on X-rays before and after the Lapidus operation

Angle	Preoperatively	Postoperatively
HVA	45.6° ± 20.5 (range 25.0° to 77.0°)	24.6° ± 14.4° (range 2.1° to 36.0°)
IMA	16.9° ± 3.8° (range 8.5° to 28.5°)	9.8° ± 5.3° (range − 2.0° to 27.8°)
TASA	3.8° ± 8.5° (range − 17.0° to 26.0°)	9.9° ± 6.9° (range − 5.6° to 30.3°)
PASA	20.5° ± 7.5° (range 3.3° to 40.0°)	19.4° ± 6.9° (range 2.6° to 36.9°)
DASA	1.5° ± 4.0° (range − 9.0° to 20.0°)	3.4° ± 5.2° (range − 13.5° to 15.0°)

*HVA – hallux valgus angle, IMA – intermetatarsal angle, TASA – tangential angle to the second axis, DASA – distal articular set angle; values mean ± standard deviation (range)*

**Fig. 2:**
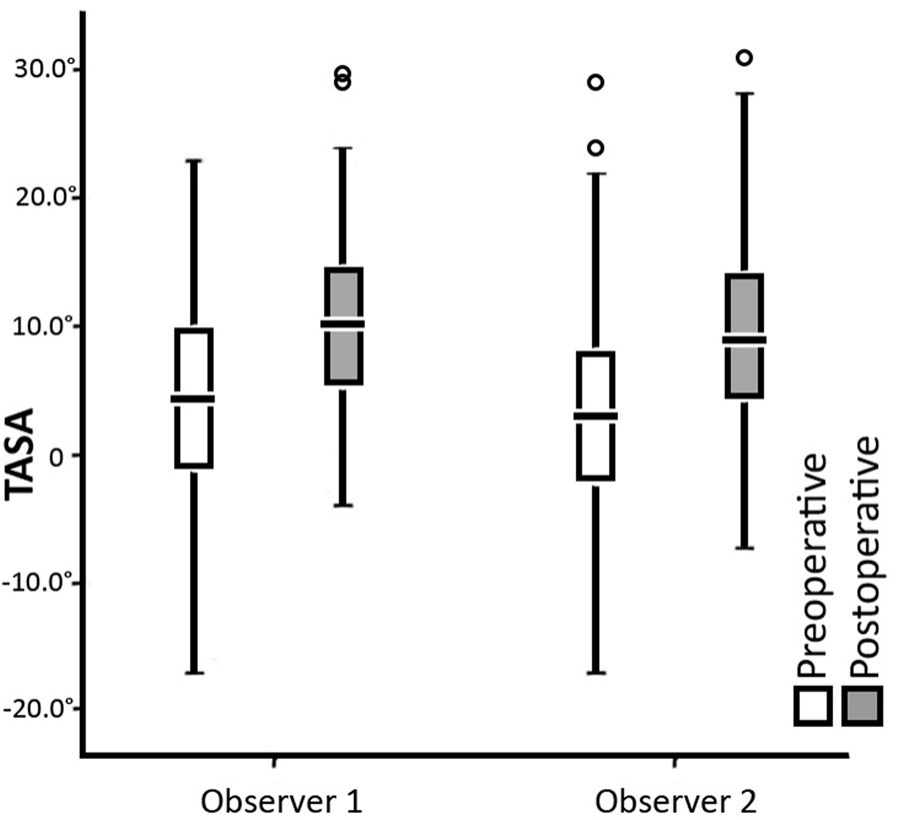
The distribution of the tangential angle to second axis (TASA) values recorded by two observers. *White – preoperative values; gray – postoperative values*

We found out that there was a significant change of the TASA angle when comparing preoperative to postoperative radiographs (*p < 0.001, η*^*2*^ *= 0.361)*. The average change was 6.1° ± 6.9°. The distribution of TASA angle values recorded by two observers is displayed in Fig. 2.

**Fig. 3:**
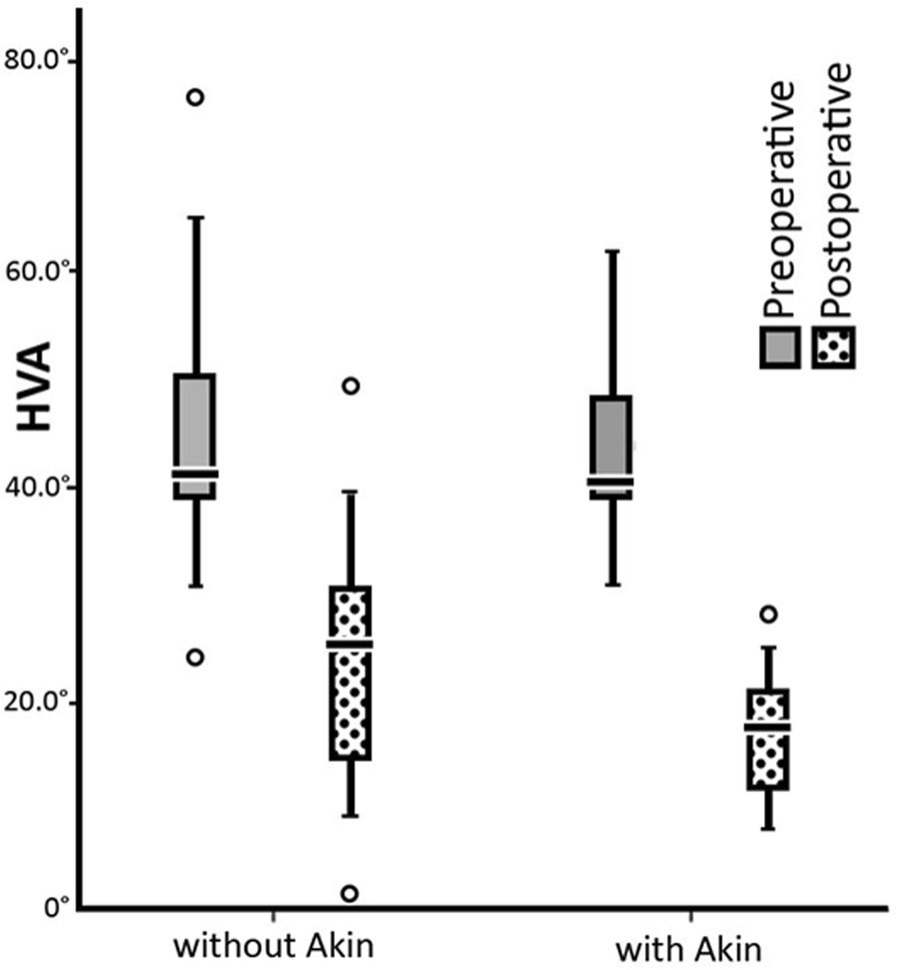
The distribution of HVA values before and after surgery. *Patients who had the Lapidus procedure only are shown on the left side. Patients who had a combination of the Lapidus and Akin procedures are shown on the right side. Gray area denotes preoperative values; dotted area denotes postoperative values*

We also compared two subgroups of patients according to the surgical procedure used. The first group underwent Lapidus arthrodesis only and the second one had Lapidus procedure combined with Akin osteotomy. Results of HVA and TASA measurements in both groups before and after surgery are displayed in Table 2. The distribution of HVA angle values recorded for Akin+/Akin- groups of patients is shown in Fig. 3.

**Table 2:** Angles of interest measured on X-rays before and after Lapidus procedure

Angle	Preoperatively		Postoperatively	
	without Akin	with Akin	without Akin	with Akin
HVA	45.6° ± 20.5° (range 25.0° to 77.0°)	42.7° ± 20.7° (range 28.0° to 56.0°)	26.2° ± 15.1° (range 2.1° to 36.0°)	17.3° ± 11.9° (range 6.6° to 26.6°)
TASA	3.7° ± 8.5° (range − 17.0° to 23.4°)	4.7° ± 8.0° (range − 6.0° to 23.5°)	9.7° ± 6.5° (range − 5.6° to 27.8°)	10.9° ± 8.5° (range − 2.5° to 30.1°)
DASA	1.5° ± 4.1° (range − 9.0° to 20.0°)	1.1° ± 3.8° (range − 5.8° to 7.5°)	4.6° ± 4.1° (range − 8.3° to 15.0°)	-4.3° ± 4.8° (range − 13.5° to 5.9°)

*HVA* – hallux valgus angle, *TASA* – tangential angle to second axis, *DASA* – distal articular set angle

values: mean ± standard deviation (range)

**Fig. 4:**
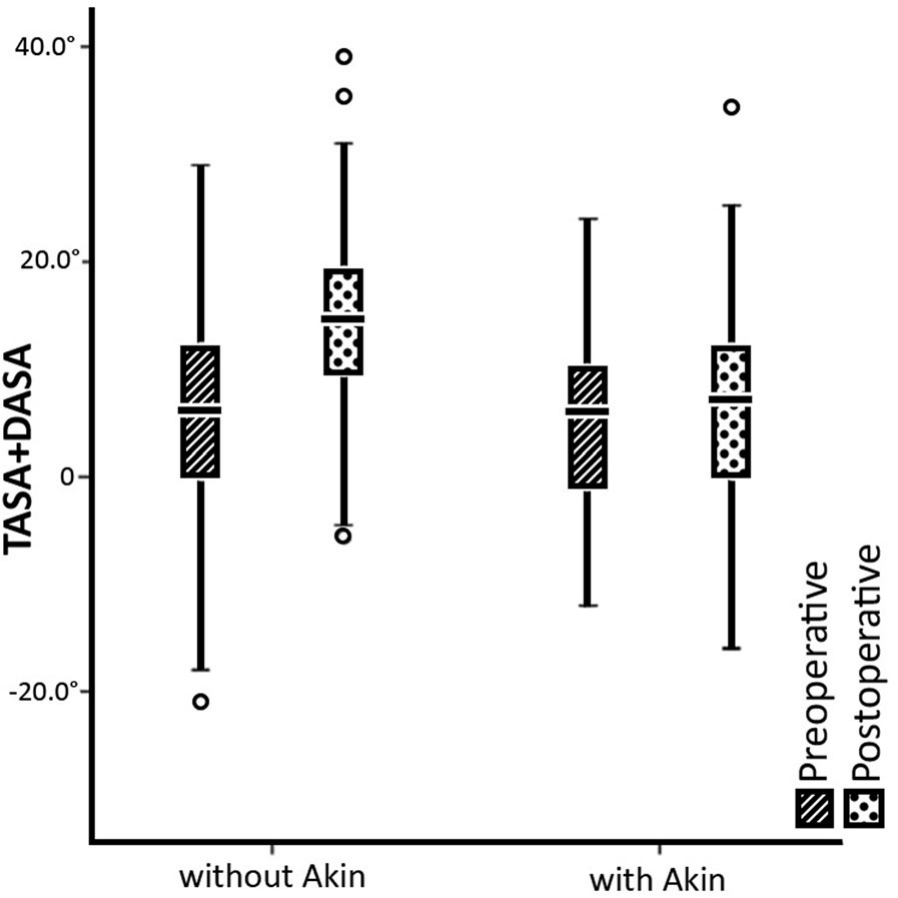
The distribution and comparison of sums of TASA and DASA angles. *Patients who had the Lapidus procedure only are shown on the left side. Patients who had a combination of the Lapidus and Akin procedures are shown on the right side. Striped area denotes preoperative values; dotted area denotes postoperative values*

The Fig. 4 shows distribution of TASA and DASA sums. The mean value of these angles total for a Lapidus only procedure was 5.2° ± 9.3° before and 14.2° ± 7.8° after the operation. In the group of patients, where the additional Akin osteotomy was used, the mean value was 5.3° ± 8.4° before the operation and 6.9° ± 10.2° after the two procedures. A significant difference, comparing these two groups, was found (*p = 0.005, η*^*2*^ *= 0.075*). The mean difference in values (TASA+DASA) between the two groups was 7.3° of extra correction in favour of the group with an additional Akin osteotomy.

## Discussion

Bunion surgery is a common part of the orthopaedic practice. The reduction of hallux valgus angle (HVA) using various types of osteotomies is one of the main purposes of the surgery. The proper alignment of the hallux is achievable only by a combination of soft tissue and bony procedures changing position of articular surfaces.

The Lapidus procedure is an effective operation that can reduce deformity of the forefoot and improve position of the hallux. The surgery allows not only a reduction of the first intermetatarsal angle, but, more importantly, it can change the rotation and length of the first metatarsal [4–10]. The procedure has even disadvantages. The operative technique is demanding for the skill of the surgeon, since the position of the arthrodesis must address the deformity in all 3 dimensions. Especially important is the rotational part of the deformity. That leads to a lower reproducibility of the surgery results when comparing the work of more surgeons, as the personal experience with the arthrodesis positioning is crucial and the learning curve is longer. Beside that, there is an insufficient control over the first metatarsal’s distal articular surface position, if fluoroscopy imaging is not used peroperatively.

The reduction of IMA, which is also usually pursued by any type of reconstruction surgery, may lead to an increase in TASA value as well. Theoretically, in normal conditions the formula TASA = PASA-IMA is valid [14]. The PASA angle describes the orientation of the distal articular surface in relation to the first metatarsal shaft axis. The angle is not changed by the Lapidus type arthrodesis. In our study, the mean TASA value increase was 6.1 ± 6.9°, while there was a mean decrease of 7.1° in IMA values. The average difference was 1° and the median was 0°. The TASA angle will increase equally to the decrease of IMA after the Lapidus procedure. Surgeons should expect it especially when the TASA is positive before the operation.

The outcome of the operation depends not only on IMA reduction, but also on the rotation of the first metatarsal. The pronation of the metatarsal is an integral and logical by-product of every hallux valgus deformity and its precise correction (derotation) is an essential part of the correction process. [4, 5]. Originally, we thought that the derotation of the first metatarsal would strongly influence the TASA angle. Our anticipation was, that correction of the pronation would lead to an improvement in TASA values. Contrary to that, our results showed that the derotation generally does not influence the TASA angle at all. Since there is not enough evidence in the literature about the use of TASA in the planning of surgery, our aim was to assess its usefulness in this field. We found out that there were enormous individual differences in TASA values among various patients´ cases (ranging from − 20° to 26°). We do not recommend using this method in precise preoperative measurements.

The DASA and PASA compile a set of angles. The set of these angles i.e. articular surfaces position is important for correction of the hallux valgus deformity. The size of DASA angle influences not only the mechanical axis of the toe, but even takes an effect in the action of extrinsic muscles as well [15]. The lower the DASA value is after surgery, the better deformity correction was achieved [16]. The DASA should not be influenced by Lapidus operation. Nevertheless, we recorded a change in DASA values before and after surgical procedure. The average change was 2.5° without Akin procedure and 7.0° using the procedure. The significant change in cases without using a phalangeal osteotomy was due to an initial pronation of the toe (as a part of the hallux valgus deformity). The change was a consequence of restoring the physiological position of first metatarsal in coronal plane.

In our study, we evaluated the influence of an additional Akin procedure on the forefoot reconstruction using Lapidus procedure. Not many current studies have assessed the hallux valgus correction results depending on the use of Akin osteotomy [17]. The sum of TASA and DASA angles was evaluated for a foot operated only with Lapidus arthrodesis or both Lapidus and Akin procedures. The Fig. 3 shows distribution and average values of the set of angles. The study demonstrates, beside other things, that if Lapidus procedure increases the TASA angle, a reduction of the DASA can improve position of the set of articular surfaces. The mean statistical difference in the correction of articular surfaces position with and without Akin osteotomy is 7.3° (TASA+DASA sums), the reduction of DASA achieved by an additional Akin procedure was an extra 8.9° (4.6° postoperatively in the Akin- group vs. − 4.3° in the Akin+ group, see Table 2). We recommend performing an additional Akin procedure in every case, where the first MTP joint soft tissue release cannot ensure a sufficient correction of hallux valgus deformity.

In our opinion, the unfavourable change of TASA by Lapidus arthrodesis can’t be influenced by any osteotomy at the level of first MTC joint or proximal part of the first metatarsal. Another possible solution than Akin operation would be adding of a distal metatarsal osteotomy (e.g. Reverdin-Isham osteotomy [18]) to the first MTC joint fusion. However, this could possibly lead to the stiffness of first MTP joint.

Our radiographic study didn’t evaluate the soft tissue balance. The soft tissue balance and different amount of weight-bearing changes position of the toe and hallux valgus angle. The soft tissue procedures such as lateral release of first MTP joint to restore physiological position of the sesamoids or release of long extensor hallucis tendon were used as a complementary procedure.

An example of the use of our measurements can be seen in above mentioned illustrations. The TASA angle value (Fig. 1a) is negative (− 10°). After the reduction of IMA, it switches to positive values (2°). Equation TASA = PASA-IMA is valid and PASA value wasn’t changed by surgery. The IMA was reduced by 12°. The PASA and DASA angles preoperatively are approx. 30° (Fig. 1a). The PASA value is more or less the same after the arthrodesis (Fig. 1b). The DASA is reduced nearly to 0° by an Akin osteotomy. If only a lateral release was used as a complementary procedure to Lapidus arthrodesis, the final position of the hallux under full weight-bearing would be unsatisfactory. Therefore, an additional Akin osteotomy was used to improve the axis of the hallux. Surgeons should be careful especially in a case where TASA angle is positive before the operation.

There are several limitations of our study – we have not evaluated tibial sesamoid position (TSP) on pre- and postoperative radiographs. This variable is important for the clinical outcome of any reconstruction surgery. The present study was aimed predominantly on the articular surface position before and after the surgery and on the role of TASA angle in hallux valgus surgery assessment and planning, not on the correction success rate. On the other hand, we admit, that the inclusion of TSP measurement would bring even deeper insight into the surgery impact.

Another possible limitation of the study is the variety of fixation techniques used – the arthrodesis of first MTC joint was done mostly with memory staples but in some cases also using 2 or 3 screws or a plate. Additionally, the peroperative fluoroscopic control of the desis position was not available in all cases. These facts decrease the homogeneity of the studied group of patients and the reproducibility of the surgery results.

Further research is also needed to compare the Lapidus osteotomy with Akin procedure before and after surgery using subjective outcome scoring systems (AOFAS Forefoot score etc.).

## Conclusions

The results of our X-ray analysis confirm the hypothesis of an unfavourable lateral inclination of the articular surface of first metatarsal head after the Lapidus procedure. The mean worsening of TASA angle after performing of Lapidus operation is 6.1° ± 6.9°. The significant deterioration after the operation corresponds with reduction of IMA and it is not caused by the derotation of the first metatarsal. The Akin osteotomy of the proximal phalanx is a suitable complement to the Lapidus arthrodesis and improves the articular set (position of the articular surfaces) of the first MTP joint.

## Abbreviations

AOFAS: American Orthopaedic Foot & Ankle Society
DASA: Distal articular set angle
HVA: Hallux valgus angle
IMA: Intermetatarsal angle
MTC: Metatarsocuneiform
MTP: Metatarsophalangeal
PASA: Proximal articular set angle
TASA: Tangential angle to the second metatarsal axis
TSP: Tibial sesamoid position
